# Genes and Gut Bacteria Involved in Luminal Butyrate Reduction Caused by Diet and Loperamide

**DOI:** 10.3390/genes8120350

**Published:** 2017-11-28

**Authors:** Nakwon Hwang, Taekil Eom, Sachin K. Gupta, Seong-Yeop Jeong, Do-Youn Jeong, Yong Sung Kim, Ji-Hoon Lee, Michael J. Sadowsky, Tatsuya Unno

**Affiliations:** 1 Faculty of Biotechnology, School of Life Sciences, SARI, Jeju National University, Jeju 63243, Korea; nakwon3070@gmail.com (N.H.); sachinkumargupta1721@gmail.com (S.K.G.); 2 Subtropical/tropical Organism Gene Bank, Jeju National University, Jeju 63243, Korea; taekil7@hanmail.net; 3 Microbial Institute for Fermentaion Industry (MIFI), Sunchang, Jeonbuk 595804, Korea; khs8706@naver.com (S.-Y.J.); jdy2534@korea.kr (D.-Y.J.); 4 Department of Gastroenterology, Wonkwang University Sanbon Hospital, Gunpo 435040, Korea; wms89@hanmail.net; 5 Department of Bioenvironmental Chemistry, Chonbuk National University, Jeonju 54896, Korea; jhlee2@jbnu.ac.kr; 6 BioTechnology Insittute, University of Minnesota, St. Paul, MN 55108, USA; sadowsky@umn.edu; 7 Department of Soil, Water, and Climate, University of Minnesota, St. Paul, MN 55108, USA; 8 Department of Plant and Microbial Biology, University of Minnesota, St. Paul, MN 55108, USA

**Keywords:** butyrate synthesis, gut microbiota, gut dysbiosis, metagenomics, mucin

## Abstract

Unbalanced dietary habits and gut dysmotility are causative factors in metabolic and functional gut disorders, including obesity, diabetes, and constipation. Reduction in luminal butyrate synthesis is known to be associated with gut dysbioses, and studies have suggested that restoring butyrate formation in the colon may improve gut health. In contrast, shifts in different types of gut microbiota may inhibit luminal butyrate synthesis, requiring different treatments to restore colonic bacterial butyrate synthesis. We investigated the influence of high-fat diets (HFD) and low-fiber diets (LFD), and loperamide (LPM) administration, on key bacteria and genes involved in reduction of butyrate synthesis in mice. MiSeq-based microbiota analysis and HiSeq-based differential gene analysis indicated that different types of bacteria and genes were involved in butyrate metabolism in each treatment. Dietary modulation depleted butyrate kinase and phosphate butyryl transferase by decreasing members of the Bacteroidales and *Parabacteroides*. The HFD also depleted genes involved in succinate synthesis by decreasing *Lactobacillus*. The LFD and LPM treatments depleted genes involved in crotonoyl-CoA synthesis by decreasing *Roseburia* and *Oscilllibacter*. Taken together, our results suggest that different types of bacteria and genes were involved in gut dysbiosis, and that selected treatments may be needed depending on the cause of gut dysfunction.

## 1. Introduction

Butyrate is a bioactive compound that inhibits histone deacetylase (HDAC). Inhibition of HDAC has been studied in mammalian cells, and inhibits proliferation, induction of differentiation, and induction/repression of gene expression. However, inhibition of HDAC activity affects the expression of only ~2% of mammalian genes [1]. Butyrate is also known to activate G-protein-coupled receptors (GPCR), by which it regulates diverse body functions. In addition, butyrate has antitumor activity by activating GPR43 [2]. Recent gut microbiome studies have revealed that butyrate also serves as an energy source to gut epithelial cells. Donohoe et al. [3] reported that gut microbiota influences the host’s metabolic functions by regulating the abundance of mRNA and proteins involved in metabolism, especially the tricarboxylic acid (TCA) cycle. Deprived energy status leads to autophagy in the colon. In contrast, feeding mice with the butyrate producing bacterium, *Butyrivibrio fibrisolvens* increased ATP and restored autophagy to the normal level. Butyrate intake as a dietary supplement was also found to improve insulin sensitivity in mice [4], counteract adverse effects caused by a high-fat diet [5] and was useful in treating Type 1 diabetes by improving beta-cell proliferation and glucose homeostasis [6].

One of the possible underlying mechanisms by which butyrate improves metabolic disorder is by production of mucin. Mucins are high molecular weight glycoproteins produced by goblet cells that resides throughout the gastrointestinal (GI) tract. Consequently, a large part of the colon is covered with mucins. While this mucosal layer acts as an innate defense system against bacterial infection, some species of gut bacteria can colonize the mucous layer. It has been reported that *Akkermansia muciniphila* can alter the host’s metabolism, reversing effects of obesity, diabetes, and inflammation [7]. Other bacteria that colonize the inside of the mucosal layer include *Bifidobacterium*, well-known probiotic strains that are reported to improve the host’s metabolism [8]. In addition, *Bacteroides* and *Ruminococcus* are known butyrate-producing bacteria that are also found within mucosal layers [9].

In vivo studies are often conducted to study roles of gut microbiota in dysbiosis. In general, two types of gut dysbiosis induction methods have been used with mice models. One uses a dietary-based gut dysbiosis induction method, whereby high-fat diets (HFD) and low-fiber diets (LFD) are used as to represent unhealthy and unbalanced dietary habits. The other uses drugs to cause gut dysbiosis. Loperamide (LPM) is frequently applied to slow gastrointestinal (GI) motility, and consequently induces constipation in mice.

Previous studies have reported that these three treatments (HFD, LFD, and LPM) shift gut microbiota and decrease concentrations of luminal butyrate [10,11,12]. Restoration of butyrate synthesis in the colon was shown to improve the host physiological status [4,5]. Since the primary source of butyrate in humans comes from bacterial fermentation of carbohydrates in the colon, having abundant butyrate-producing bacteria and their substrate producing bacteria (i.e., lactic acid producing bacteria) could be a key to maintain optimal amounts of butyrate in the gut [13]. Various types of polysaccharides can be fermented by different butyrate producing bacteria [14], and amount of luminal butyrate depends on both the types of butyrate producers and polysaccharides present in the gut. The butyrate producers are mostly strict anaerobes, but are phylogenetically diverse [14], and most remain uncultured [15]. Thus, the abundance of the butyrate producing bacteria may need to be estimated by functional-based approaches [16].

Previously, four different metabolic pathways were proposed for butyrate synthesis by gut bacteria: starting from glutarate, acetyl-CoA, lysine, and succinic acid [17,18]. Since various types of bacteria are involved in these butyrate synthesis routes, and gut dysbiosis decreases luminal butyrate by inhibiting the activities of specific bacteria, restoration of luminal butyrate synthesis may require different treatments depending on the cause(s) of dysbiosis. In the present study, we investigated the bacteria and genes involved in butyrate synthesis using a mouse model of gut dysbiosis. Although not inclusive of all mechanisms of gut dysbiosis associated with butyrate reduction, the results in this study will provide a fundamental understanding of the inhibition of luminal butyrate production by three different causes of gut dysbiosis.

## 2. Materials and Methods

### 2.1. Experimental Design for Animal Studies

The Jeju National University Institutional Animal Care and Use Committee approved the animal experiments (JNU-IACUC; Approval Number 2016-0045). Five-week-old female ICR mice (Orient Bio Inc., Seungnam, Korea) were housed at 22 °C, with a relative humidity of 50–55% and a 12 h light/dark cycle. Mice were housed in separate cages and supplied with food and water ad libitum. After one week of acclimation, a three-week feeding trial was conducted. Animals were divided into four groups (*n* = 10 per group): control diet (CTL; 5L79, Orient Bio Inc., Seungnam, Korea), 45% HFD (D12451, Research Diets, New Brunswick, NJ, USA), LFD (Crea Japan, Inc., Shizuoka, Japan) containing 0.1% crude fiber, and control diet with 1.5 mg kg^−1^ of orally injected LPM daily on days 11–21. Two mice in LPM group died during the feeding trial, thus, eight mice were used for analyses. The calorie content of the diets used in this study are provided in Table S1.

### 2.2. Mucosal Studies

Animals were sacrificed on day 21 for histopathology study. Mucosa thickness was measured according to the method described by Kim et al. [19]. Briefly, the transverse colon was collected by dissection, sliced to a rectangle, fixed with 5% formalin for 48 h, embedded in paraffin wax, and then sectioned into 3 μm thick slices. Fixed specimens were subsequently deparaffinized with xylene, rehydrated, rinsed with distilled water, and stained with an alcian blue. Stained colon sections were observed by light microscopy (Olympus IX73, Tokyo, Japan) and the mucosa thickness was measured using cellSens (Olympus, Tokyo, Japan).

### 2.3. Butyrate Quantification

Butyrate in the cecum were measured by gas chromatography on a GC-2010 Plus gas chromatograph (Shimazu, Kyoto, Japan) equipped with a flame ionization detector. Cecum samples were obtained at the end of the feeding trials, immediately frozen in liquid nitrogen, and stored at −80 °C until used. Frozen cecum was homogenized in distilled water for 10 min. The pH was adjusted to 3 with 1 M HCl, and incubated for 10 min at room temperature with frequent agitation. Samples were centrifuged at 5000× *g* for 20 min at 4 °C, and the supernatant was stored at −20 °C. Gas chromatography was done using a fused-silica capillary column (30 m × 0.25 mm) coated with 0.25 μm thick free fatty acid phase film (DB_FFAP 122-3232, J&W Scientific, Agilent Technologies Inc., Santa Clara, CA, USA). Helium was supplied as the carrier gas using a linear velocity mode at 22.2 cm sec^−1^. The injection and detector ports were maintained at 200 °C and 240 °C, respectively. Glass wool was inserted in the liner of the splitless injection port. The oven temperature was initially set to 80 °C, then gradually increased by 6.13 °C min^−1^ to 120 °C, 20 °C min^−1^ to 150 °C, and 6.13 °C min^−1^ to 205 °C. There were 1 min intervals between the shifts, and the oven temperature was then maintained at 205 °C for 2 min. Flow rates were 40, 400, and 80 mL min^−1^ for hydrogen, air, and helium, respectively.

### 2.4. Fecal Microbiome Analysis

Feces were collected on days 7 and 20. Bacterial DNA was extracted using the PowerFecal DNA isolation kit (MOBIO Laboratories Inc., Carlsbad, CA, USA). For microbial community analysis, the V4 region of the 16S rRNA gene was amplified from fecal DNA, and a library for Illumina Miseq (250 bp × 2) was constructed with two-step PCR. For functional gene investigation, three fecal DNA samples collected at day 20 were randomly selected from each group and sequenced using Illumina HiSeq 4000 (2 × 150 bp). Both sequencing methods were performed at Macrogen Inc. (Seoul, Korea). MiSeq output was analyzed using MOTHUR [20]. The differential abundance test was performed with LEfSe [21] and community types were estimated using Dirichlet multinomial mixture (DMM) model [22], which employs probabilistic modelling to cluster microbial communities. This method was previously used to define enterotypes in the human gut [23]. Outlier samples were removed prior to analysis, based on cluster analysis using the Yue and Clayton measure of dissimilarity.

HiSeq outputs were trimmed, assembled, and annotated with the EDGE pipeline [24], using IDBA_UD for assembly [25], BWA [26] for contig abundance estimation and annotation, and Prodigal [27] for structural gene prediction. KEGG orthology (KO) assignment was performed using KAAS [28], and differential KO genes were identified by MetaPath [29]. Operational taxonomic unit (OTU)-based network analysis was performed with Cytoscape, using an organic yFile layout. Sequence data used in this study were deposited to the Short Read Archive (SRA) with project number PRJNA394775.

### 2.5. Statistical Analysis

Bioparameters were compared by using analysis of variance, with Tukey’s range and Duncan’s multiple range tests. Analysis of molecular variance [30] (AMOVA) was used to evaluate significant differences between gut microbial communities. Pearson correlation analysis was used to correlate taxa with microbial community shifts.

## 3. Results

### 3.1. Bioparametric and Histological Changes

**Figure 1:**
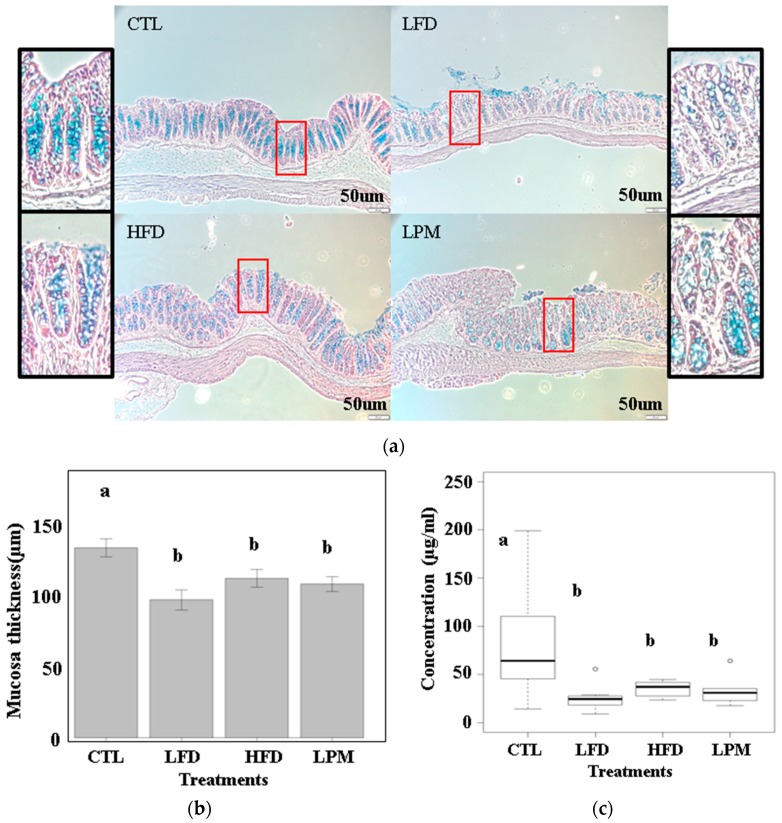
Mucosa thickness and concentration of butyrate: (**a**) histological images of intestinal tissue; (**b**) mean values (*n* = 3) of mucosa thickness; and (**c**) concentration of cecum butyrate. Different letters on bars indicate significant difference (*p* < 0.05) according to the Duncan’s multiple range test.

Throughout the three-week feeding trial, no significant body weight differences were observed among all groups. The HFD- and LFD-fed mice had significantly lower food intake and lower fecal output per gram of feed (*p* < 0.05), whereas the LPM treatment did not affect food intake or fecal output (Table S2). This difference was observed starting on the first day of the feeding trial (Figure S1). Results in Figure 1 show that HFD, LFD, and LPM decreased amount of mucin in goblet cells (Figure 1A). Significant decrease in the thickness of mucosa (*p* < 0.05) (Figure 1B) and increase in the amount of cecum butyrate (*p* < 0.05) (Figure 1C) were also observed. Other images and length of mucosa thickness were summarized in Figure S2.

### 3.2. Gut Microbial Community Comparison

A total of 134,266 MiSeq reads and 99,992,814 HiSeq reads were obtained in order to investigate bacterial community differences across the groups used in this study (Table S3). A rarefaction curve confirmed that adequate reads were obtained for microbial community analysis (Figure S3). Comparison of ecological indices indicated that the HFD and LFD groups had significantly decreased species richness (*p* < 0.05), whereas no significant differences in species evenness were observed (Figure S4). The LPM treatment did not have any statistical effect on species richness or evenness. 

**Figure 2:**
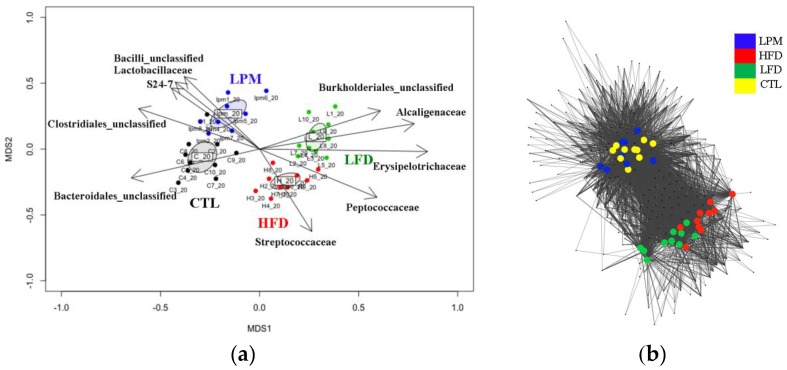
Comparison of mice gut microbiota across different treatments: (**a**) non-metric multidimensional scaling analysis; and (**b**) network analysis with operational taxonomic units (distance = 0.03).

Non-metric multidimensional scaling (NMDS) analysis demonstrated that all induction methods clearly shifted the gut microbiota (*p* < 0.001), and network analysis clearly separated the HFD and LFD groups from the CTL and LPM groups (Figure 2).

Results in Figure 2 show that HFD- and LFD-diets shifted the gut microbiota by altering both the species present and their abundance, whereas the LPM treatment only changed the abundance of existing species. Heatmap cluster analysis, done based on taxonomic composition, allowed clear separation of samples at the family level, but showed no significant differences at the phylum level (Figure S5). Therefore, correlation analysis between family level taxonomic composition and NMDS plots were analyzed.

Results in Figure 2A show that the HFD- and LFD-diet groups contained significantly decreased (*p* < 0.05) abundance of the unclassified family belonging to the phylum *Bacteroidetes* and increases in members of the families *Erysipelotrichaceae* and *Alcaligenaceae.* The abundance of members of the family S24-7 was significantly (*p* < 0.01) and positively correlated with the shift in gut microbiota caused by feed LPM.

DMM bacterial community type analysis divided the samples into two groups: CTL–LPM and HFD–LFD at the genus level. The top 10 genera responsible for this separation are shown in Table 1.

**Figure 3:**
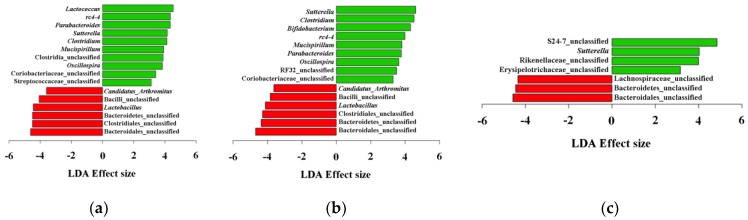
Analysis of differentially abundant genera. Red bars indicate control samples and green bars indicate high-fat diets (**a**); low-fiber diets (**b**); and loperamide administration (**c**).

Diet treatment increased *Bacteroides*, *Clostridium* (Erysipelotrichaceae), *Sutterella*, *Parabacteroides*, and *rc4-4*, while decreasing *Lactobacillus* and unclassified genera belonging to Clostridiales, Bacteroidales, Bacilli, and S24-7. DMM did not show community type difference between CTL and LPM. To investigate difference between CTL samples and each treatment, we conducted LEfSe test to identify differentially abundant genera (Figure 3).

LEfSe analyses showed significant increases in *Sutterella* and decrease in unclassified genera in Bacteroidales and Bacteroidetes in all treatments (*p* < 0.05). HFD and LFD increased *Mucispirillum*, *Parabacteroides*, *Oscillospira*, *Clostridium*, and *rc4-4*, while decreasing *Lactobacillus*, *Candidatus Arthromitus*, and unclassified genera of Clostoridiales and Bacilli (*p* < 0.05). *Lactococcus* in HFD mice and *Bifidobacterium* in LFD mice were also significantly increased (*p* < 0.05).

### 3.3. Analysis of Genes Involved in the Butanoate Metabolism Pathway

The analysis of differentially abundant KEGG reaction modules indicated that 19 genes and three KEGG entries were related to butyrate synthesis. These genes and entries are mapped to KEGG butanoate metabolism map (kegg00650) and summarized in Figures S7–S9 for HFD, LFD, and LPM, respectively. When compared to CTL mice, the HFD mice were depleted of *buk* (420 TPM) and *ptb* (399 TPM), followed by *gabD* (38 TPM; Table S4), but were enriched with 2.3.1.54 (2105 TPM) and *adhE* (1121 TPM), followed by BDH (443; Table S5). Similarly, the LFD mice were depleted of *buk* (372 TPM), and *ptb* (351 TPM), followed by *paaH* (314 TPM; Table S6), and enriched with 2.3.1.54 (2077 TPM), followed by BDH (285 TPM) and *atoD* (205 TPM; Table S7). Lastly, the LPM mice were depleted of *adhE* (599 TPM), ACAD (515 TPM), and *paaH* (459 TPM), followed by *crt* (407 TPM) (Table S8) and enriched with *ptb* (1475 TPM) and *buk* (1460 TPM), followed by *gabD* (165 TPM) (Table S9).

The *buk* and *ptb* genes were depleted in both the HFD and LFD groups, and were classified to Bacteroidales and *Parabacteroides*. In contrast, these genes were enriched in the LPM group and classified to Bacteroidales and *Bacteroides*. The *gabD* gene, which was depleted by the HFD, but enriched by LPM, was classified to *Lactobacillus*.

The HFD and LFD treatments particularly enriched genes involved in acetyl-CoA synthesis from pyruvate (2.3.1.54). While these genes were classified to diverse taxa, such as Bacteroidales and Clostridiales in the HFD group, they were mainly classified to *Bacteroides* in the LFD group. The LFD and LPM treatments both depleted *paaH*, which was classified to *Oscillibacter valericigenes* and *Roseburia hominis*. In this study, differential gene mapping to lysine degradation (map00310) did not appear to be involved in butyrate synthesis (data not shown).

## 4. Discussion

### 4.1. Duration of Feeding Trial for Reduction in Butyrate Synthesis

We conducted a feeding trial for three weeks to investigate the differences across the treatment groups used in this study. Shifting of gut microbiota by diet usually takes no longer than a week [31], and we also observed a significant gut microbiota shift at day 7 (*p* < 0.001), which was maintained for two additional weeks (Figure S6). Therefore, the three weeks was more than adequate to capture microbial shifts caused by the diets.

We also observed significant decrease in mucins, mucosa thickness, and butyrate in the gut. It has been reported that longer treatments of diet-induced dysbiosis (5 and 13 weeks for LFD and HFD, respectively) eventually causes physiological changes such as obesity and constipation [32,33]. Since our focus is on butyrate synthesis, a longer feeding trial would likely involve other factors leading to dysbiosis.

### 4.2. Mechanisms in Butyrate Reduction Induced by HFD, LFD, and LPM Treatments

SCFAs (short chain fatty acids) are byproducts of bacterial carbohydrate fermentation in the gut. The concentrations of luminal SCFAs differ depending on the bacteria and carbohydrates present in the gut. Among intestinal bacteria, *Eubacterium*, *Roseburia*, and *Faecalibacterium* are known SCFA producers [34], while *Bifidobacterium* and *Lactobacillus* are the substrate providers [35]. Brown et al. [13] suggested that optimal butyrate concentration in gut can be maintained by having abundant butyrate-producing bacteria and lactate-producing bacteria. Moreover, Bacteria in the gut are known to communicate by syntrophic interactions (cross-feeding) [36], thus, other bacteria may also play important roles in butyrate production.

#### 4.2.1. Butyrate Reduction Mechanism(s) Induced by HFD

In this study, we observed significant loss of *Lactobacillus* in HFD samples, and taxonomic classification of contigs indicated the depletion of *gabD* is mainly due to the loss of *Lactobacillus* (Table S4), resulting in decreased synthesis of butyrate from succinate. Moreover, HFD decreased the abundance of the butyrate producers *Parabacteroides* and *Bacteroides* (in the Bacteroidales) that contain the *buk* and *ptb* genes. Conversely, HFD increased the Bacteroidales, especially *Bacteroides*, who can convert pyruvate into acetyl-CoA (KEGG 2.3.1.54). Acetyl-CoA can be subsequently metabolized to butyrate. In this case, however, depletion of the *buk* and *ptb* genes did not allow for butyrate synthesis. The unused acetyl-CoA indirectly increases fat storage by feedback inhibition. The HFD treatment also increased the relative abundance of *Lactococcus*, a known probiotic species [37]. In contrast, many obese people are colonized with *Lactococcus* [38], suggesting that probiotic effects of *Lactococcus* are host, or bacterial species- or strain-specific. These *Lactococcus* species with aldehyde-alcohol dehydrogenase gene *adhE* are known to convert substrates into alcohol rather than lactate [39], further reducing syntrophically-produced lactate. Lastly, the HFD also increased a member of the genus *rc4-4*, belonging to the phylum Firmicutes. This genus is known as a diet-induced, obesity-associated bacterium [40], but its function has not been reported.

#### 4.2.2. Butyrate Reduction Mechanisms Induced by LFD

The LFD also depleted butyrate producers containing *buk* and *ptb* genes. LEfSe analysis showed an increase in *Bifidobacterium*, a known lactate producer, by LFD treatment (Figure 3B). Functional gene analysis, however, showed the increase of the *Bifidobacterium* is related to the increase in its pyruvate formate-lyase (KEGG 2.3.1.54) activity (Table S7). In this case, pyruvate is used for acetyl-CoA production rather than lactate production [41], although large part of the acetyl-CoA production was likely performed by members of the *Bacteroides* and *Parabacteroides*.

#### 4.2.3. Butyrate Reduction Mechanisms Induced by LPM

In this study, we did not observe gut dysmotility in the LPM treated mice. In addition, gut microbiota shifts in the LPM-treated mice were smaller compared to those of the HFD and LFD treated mice. Nevertheless, the LPM treatment shifted gut microbiota by increasing species of the S24-7 family and members of the genus *Sutterella*, consistent with a previous report by Touw et al. [42]. The family S24-7 is a member of uncultured Bacteroidales, and contain commensal intestinal bacteria of homeothermic animals, particularly mice. However, the metabolic functions of these species are still unknown [43]. Unlike HFD and LFD, the LPM treatment enriched *ptb* and *buk*. However, LPM depleted four KEGG reaction modules (*adhE*, ACADS, *paaH* and *crt*) possessed by unclassified bacteria (Table S8). All of these enzymes are necessary to utilize acetyl-CoA to produce crotonoyl-CoA, an intermediate metabolite for butyrate, except for *adhE*. Therefore, the increase in *ptb* and *buk* genes does not likely lead to an increase in the production of butyrate.

### 4.3. Other Bacteria Involved in Gut Dysbiosis

In all treatments, we found an increase in *Sutterella* and *Clostridium* (family Erysipelotrichaceae) and a decrease in *Anaeroplasma* (Figure 3). The family Alcaligenaceae (genus *Sutterella*) can adhere to epithelial cells, and is associated with autism, Downs syndrome, and inflammatory disease [44,45]. The family *Erysipelotrichaceae* is known to be associated with inflammation-related gastrointestinal diseases, although a large part of its functions are still unknown [46]. The decrease in the mucosal thickness induced by the loss of luminal butyrate may have allowed the increase of *Sutterella* and Erysipelotrichaceae, since they colonize epithelial cells. A decrease in *Anaeroplasma* has been reported to be associated with fecal hardness [47] and diet-induced obesity [48], suggesting that members of this genus negatively correlate with gut dysbiosis. Diet-induced gut dysbiosis has been reported to increase the abundances of *Mucispirillum*, *Parabacteroides*, *Oscillospira*, and *Anaerotruncus. Mucispirillum* colonizes mucosal layers in rodents, and is associated with gut inflammation [49]. Loy et al. [50] suggested that *Mucispirillum* may modify mucosal gene expression in the host. *Parabacteroides* is also known to be associated with diet-induced obesity. Lecomte et al. [51] reported that *Parabacteroides* is one of the major succinate producers in the gut, and is also associated with obesity. *Oscillospira* is anaerobic bacteria that has been difficult to culture. *Oscillospira* has been reported to be capable of utilizing host glycans, but is not a fiber degrader. Interestingly, this bacterium increases with consumption of a high-fat diet, and it has been known to be associated with leanness in human [52]. Due to the difficulties in growing this bacterium, Gophna et al. [53] used a metagenomics approach to infer its function. They reported that it may be a butyrate producer, and is associated with leanness and negatively correlated to intestinal inflammation, which contradicts our results. Further studies are required to understand the role of this bacterium. Lastly, *Anaerotruncus* have been reported to be positively correlated with bloating and abdominal pain in humans [54]. Although the role of this bacterium in mice is unknown, the increase of *Anaerotruncus* may have negative effects on health. 

In summary, we evaluated three treatment models often used to induce gut dysbiosis in mice, for effects on the structure and function of intestinal microbiota. We observed that butyrate production was inhibited in all dysbiosis models. MiSeq-based microbial community analysis indicated that these treatments shifted microbiota differently, and HiSeq-based metagenome analysis demonstrated that these treatments likely inhibited multiple butyrate biosynthetic pathways. Since our results suggest that the mechanism of butyrate inhibition may differ depending on which pathway is involved, different strategies may need to be adopted for efficiently cure gut dysbiosis.

## Figures and Tables

**Table 1:** Top 10 genera with higher differences in Dirichlet multinomial mixtures analysis.

Group	Mean			Cumulative Fraction	Genus
	Total	HFD–LFD	CTL–LPM		
CTL–LPM	6.88	4.66	9.58	0.11	Clostridiales unclassified
CTL–LPM	4.16	2.42	6.69	0.2	*Lactobacillus*
HFD–LFD	40.29	42.01	37.8	0.29	*Bacteroides*
HFD–LFD	2.17	5.11	0.99	0.38	*Clostridium* (Erysipelotrichaceae)
CTL–LPM	1.6	0.7	4.09	0.46	Bacteroidales unclassified
HFD–LFD	2.33	3.86	1.27	0.51	*Sutterella*
CTL–LPM	1.38	0.63	3.09	0.57	Bacilli unclassified
HFD–LFD	3.32	4.15	2.23	0.61	*Parabacteroides*
HFD–LFD	2.51	3.41	1.56	0.65	*rc4-4*
CTL–LPM	0.69	0.25	1.82	0.68	S24-7 unclassified

## Supplementary Materials

The following are available online at www.mdpi.com/2073-4425/8/12/350/s1. Figure S1: Time series bioparametric changes for body weight, food/water intake, and fecal output/gram of feed; Figure S2: Images and thickness (μm) of mucosa stained with alcian blue; Figure S3: Rarefaction curve analysis for CTL, HFD, LFD, and LPM samples, indicated C, H, L, and lpm, respectively. “_20” indicates “day 20” when samples were obtained; Figure S4: Comparison of ecological indices between constipated mice gut microbiota: (A) for species richness; (B) for species evenness. Different letters indicate significant difference according to Duncan’s multiple range test (*p* < 0.05). Figure S5: Taxonomic composition comparison: (A) at the phylum; (B) at the family level. Black, red, green, and blue indicate CTL, HFD, LFD and LPM, respectively; Figure S6: Comparison of gut microbiota at day 7 and day 20. C, H, L, and lpm indicate control, high fat diet, low fiber diet, and loperamide groups, respectively. Numbers after sample names indicate day of the feeding trial. Figure S7: Significantly affected KEGG reaction modules by high-fat diet treatment; Figure S8: Significantly affected KEGG reaction modules by low-fiber diet treatment; Figure S9: Significantly affected KEGG reaction modules by loperamide treatment; Table S1: Calories of feeds used in this study. Table S2: Changes in body weight, feed intake, water intake, and fecal parameters of mice at day 21. Table S3: Average number of reads, operational taxonomic units, and structural genes for MiSeq and HiSeq. Table S4: Abundance (TPM) and taxa of CTL contigs with higher differential abundance compared to HFD. Table S5: Abundance (TPM) and taxa of HFD contigs with higher differential abundance compared to CTL; Table S6: Abundance (TPM) and taxa of CTL contigs with higher differential abundance compared to LFD; Table S7: Abundance (TPM) and taxa of LFD contigs with higher differential abundance compared to CTL; Table S8: Abundance (TPM) and taxa of CTL contigs with higher differential abundance compared to LPM; Table S9: Abundance and taxa of LPM contigs with higher differential abundance compared to CTL.
