# Supplementary material for: Genes and Gut Bacteria Involved in Luminal Butyrate Reduction Caused by Diet and Loperamide
Source: Genes (Basel). 2017 Nov 28;8(12):350. doi: 10.3390/genes8120350 (PMC5748668; doi:10.3390/genes8120350)
Supplement: Supplementary file 1 [file genes-08-00350-s001.pdf]

Table S1. Calories of feeds used in this study

	Normal diet	Low fiber diet	45% High fat diet
Protein (%)	21.03	24.38	20
Carbohydrate (%)	65.30	62.19	35
Fat (%)	13.67	13.43	45
Kcal/g	4.04	4.02	4.73

TableS2. Changes in body weight, feed intake, water intake and fecal parameters of mice at day 21.

**Bioparameters**	**Treatments**			
	**Control**	**Low-fiber diet**	**High-fat diet**	**Loperamide**
Body weight (g)	27.66±0.34	26.74±0.32	26.85±0.30	26.52±0.49
Food intake (g)	6.89±047	4.78±0.37*	4.03±0.31*	6.45±0.44
Water intake (g)	7.71±0.32	7.73±0.92	6.31±0.60	7.41±0.47
Fecal output g^{-1} of food (n)	18±0.60	8.80±1.45*	8.68±1.07*	17.51±1.06

*P<0.05

Different letters also indicate significant difference based on Duncan’s multiple range test

Table S3. Average number of reads, operational taxonomic units, and structural genes for MiSeq and HiSeq

Samples	MiSeq		HiSeq	
	**Reads**	**OTUs**	**Reads**	**Genes**
CTL	35,322 (7,756)[a]	149 (20)	26,650,146 (1,164,704)	140,749 (35,318)
HFD	31,474 (5,768)	99 (9)	24,921,520 (437,432)	125,488 (9,837)
LFD	30,570 (5,937)	106 (5)	24,176,031 (1,160,224)	120,265 (22,184)
LPM	36,900 (3,128)	150 (19)	24,245,117 (2,163,476)	82,109 (3,437)

[a] Standard deviation

Table S4. Abundance (TPM) and taxa of CTL contigs with higher differential abundance compared to HFD

Lowest common ancestor	buk	ptb	gabD	ACSM
Lactobacillus	0	0	20	0
Lactobacillus johnsonii	0	0	6	0
Lactobacillus johnsonii DPC 6026	0	0	9	0
Lactobacillus reuteri TD1	0	0	3	0
Bacteroidales	362	362	0	0
Parabacteroides distasonis ATCC 8503	65	65	0	0
Bacteria	51	51	0	0
Roseburia hominis A2-183	7	7	0	0
Gammaproteobacteria	7	7	0	0
Unclassified	420	399	38	9
Total	912	891	76	9

Table S5. Abundance (TPM) and taxa of HFD contigs with higher differential abundance compared to CTL

**Lowest common ancestor**	**2.3.1.54**	**adhE**	**BDH**	**gctA**	**gctB**	**4.1.3.4**	**mhpF**
Lactococcus lactis subsp. cremoris A76	0	0	72	0	0	0	0
Lactococcus lactis subsp. cremoris NZ9000	18	0	7	0	0	0	0
Lachnoclostridium phytofermentans ISDg	0	0	7	0	0	0	0
Roseburia hominis A2-183	79	71	0	0	0	0	0
Lactococcus lactis	0	29	0	0	0	0	0
Clostridiales	130	48	0	0	0	0	0
Bacteria	3	18	0	0	0	10	0
Lactococcus lactis subsp. cremoris MG1363	0	25	0	0	0	0	0
Mycoplasma leachii PG50	0	17	0	0	0	0	0
Oscillibacter valericigenes Sjm18-20	31	8	0	10	0	0	0
Bacteroidales	449	0	0	0	0	0	0
Bacteroides	286	0	0	0	0	0	0
Thermobacillus composti KWC4	36	0	0	0	0	0	0
Lactococcus lactis subsp. cremoris KW2	31	0	0	0	0	0	0
Clostridium beijerinckii NCIMB 8052	50	0	0	0	0	0	0
Clostridium sp. SY8519	40	0	0	0	0	0	0
Firmicutes	32	0	0	0	0	0	0
Clostridium saccharoperbutylacetonicum N1-4(HMT)	27	0	0	0	0	0	0
Parabacteroides distasonis ATCC 8503	14	0	0	0	0	0	0
Peptococcaceae	10	0	0	0	0	0	0
Clostridium saccharobutylicum DSM 13864	8	0	0	0	0	0	0
Bacteroides thetaiotaomicron VPI-5482	4	0	0	0	0	0	0
Unclassified	2105	1121	443	70	33	14	14
Total	3354	1339	528	80	33	24	14

Table S6. Abundance (TPM) and taxa of CTL contigs with higher differential abundance compared to LFD

**Lowest common ancestor**	**buk**	**ptb**	**paaH**	**fabV**	**phbB**
Oscillibacter valericigenes Sjm18-20	0	0	25	0	0
Bacteria	51	51	0	34	0
Roseburia hominis A2-183	7	7	15	0	0
Bacteroidales	362	362	0	0	0
Parabacteroides distasonis ATCC 8503	65	65	0	0	0
Gammaproteobacteria	7	7	0	0	0
Unclassified	421	400	460	118	4
Total	912	891	499	152	4

Table S7. Abundance (TPM) and taxa of LFD contigs with higher differential abundance compared to CTL

**Lowest common ancestor**	**2.3.1.54**	**BDH**	**atoD**	**paaF**	**gctA**	**gctB**
Roseburia hominis A2-183	56	26	0	0	27	27
Bacteroides	1085	0	0	0	0	0
Clostridium saccharobutylicum DSM 13864	115	0	0	0	0	0
Bacteroidales	34	0	0	0	0	0
Clostridium lentocellum DSM 5427	13	0	0	0	0	0
Mageeibacillus indolicus UPII9-5	81	0	0	0	0	0
Parabacteroides distasonis ATCC 8503	11	0	0	0	0	0
Bacteria	10	0	0	26	0	0
Lactobacillus salivarius CECT 5713	22	0	0	0	0	0
Bifidobacterium	4	0	0	0	0	0
Oscillibacter valericigenes Sjm18-20	13	0	0	0	10	0
Clostridiales	114	0	0	0	0	0
Akkermansia muciniphila ATCC BAA-835	25	0	0	0	0	0
Firmicutes	50	0	0	0	0	0
Streptococcus equi subsp. zooepidemicus	12	0	0	0	0	0
Rhodanobacter denitrificans	19	0	0	0	0	0
Bacillus cytotoxicus NVH 391-98	13	0	0	0	0	0
Symbiobacterium thermophilum IAM 14863	0	0	11	0	0	0
Bacteroides vulgatus ATCC 8482	84	0	0	0	0	0
Escherichia coli APEC O78	21	0	0	0	0	0
Escherichia coli SE11	17	0	0	0	0	0
Escherichia coli W	16	0	0	0	0	0
Lactobacillaceae	12	0	0	0	0	0
Bacteroides fragilis NCTC 9343	9	0	0	0	0	0
Streptococcus anginosus C238	9	0	0	0	0	0
Fusobacterium nucleatum	8	0	0	0	0	0
Enterobacteriaceae	6	0	0	0	0	0
[Clostridium] saccharolyticum WM1	5	0	0	0	0	0
Escherichia coli	0	0	0	15	0	0
Unclassified	2077	285	205	41	29	28
Total	3940	311	216	81	66	55

Table S8. Abundance (TPM) and taxa of CTL contigs with higher differential abundance compared to LPM

Lowest common ancestor	adhE	ACADS	paaH	crt	ydiF	ACSM	mhpF	ECHS1	phbB	atoD
[Eubacterium] eligens ATCC 27750	66	0	0	0	0	9	0	0	0	0
Candidatus Arthromitus sp. SFB-rat-Yit	5	0	0	0	0	0	0	0	0	0
Ruminococcus albus 7 = DSM 20455	3	0	0	0	0	0	0	0	0	0
Roseburia hominis A2-183	19	32	15	26	0	0	0	0	0	0
Oscillibacter valericigenes Sjm18-20	0	15	25	0	8	0	0	0	0	0
Lactobacillus	30	0	0	0	0	0	0	0	0	0
[Clostridium] stercorarium subsp. stercorarium DSM 8532	9	0	0	0	0	0	0	0	0	0
Candidatus Arthromitus sp. SFB-mouse-Japan	4	0	0	0	0	0	0	0	0	0
Bacteroides	0	26	0	0	0	0	0	0	0	0
Bacteria	6	14	0	0	0	0	0	0	0	0
Thermanaerovibrio acidaminovorans DSM 6589	6	0	0	0	0	0	0	0	0	0
Firmicutes	6	0	0	0	0	0	0	0	0	0
Lactobacillus johnsonii NCC 533	5	0	0	0	0	0	0	0	0	0
Clostridiales	4	0	0	0	0	0	0	0	0	0
Clostridium sp. SY8519	0	6	0	0	0	0	0	0	0	0
Unclassified	435	422	459	381	4	0	9	5	4	3
Total	599	515	499	407	11	9	9	5	4	3

Table S9. Abundance and taxa of LPM contigs with higher differential abundance compared to CTL

**Lowest common ancestor**	**ptb**	**buk**	**gabD**	**2.3.3.10**
Lactobacillus	0	0	47	37
Lactobacillus johnsonii N6.2	0	0	0	12
Lactobacillus reuteri TD1	0	0	14	4
Lactobacillus johnsonii	0	0	72	12
Arcobacter nitrofigilis DSM 7299	0	0	4	0
Bacteroides	642	642	0	0
Roseburia hominis A2-183	10	10	0	0
Lactobacillus reuteri	0	0	0	6
Bacteroidales	242	242	0	0
Alistipes	8	8	0	0
Mycobacterium smegmatis JS623	0	6	0	0
Lactobacillus johnsonii DPC 6026	0	0	0	10
Lactobacillus johnsonii NCC 533	0	0	22	0
Lactobacillus ruminis ATCC 27782	28	28	0	0
unclassified	544	523	6	0
total	1475	1460	165	82
